# The prognostic utility of GRACE risk score in predictive cardiovascular event rate in STEMI patients with successful fibrinolysis and delay intervention in non PCI-capable hospital: a retrospective cohort study

**DOI:** 10.1186/s12872-016-0383-3

**Published:** 2016-11-08

**Authors:** Yotsawee Chotechuang, Arintaya Phrommintikul, Roungtiva Muenpa, Jayanton Patumanond, Tuanchai Chaichuen, Srun Kuanprasert, Noparat Thanachikun, Thanawat Benjanuwatra, Apichard Sukonthasarn

**Affiliations:** 1 Clinical Epidemiology Program, Faculty of Medicine, Chiang Mai University, Chiang Mai, 50200 Thailand; 2 Cardiology Division, Internal Medicine Department, Lampang Hospital, Lampang, 52000 Thailand; 3 Cardiology Division, Department of Internal Medicine, Faculty of Medicine, Chiang Mai University, Chiang Mai, 50200 Thailand; 4 Pharmaceutical Care Unit, Pharmacy Department, Lampang Hospital, Lampang, 52000 Thailand; 5 Center of Excellence in Applied Epidemiology, Faculty of Medicine, Thammasat University, Bangkok, 12121 Thailand; 6 Cardiac catheterization laboratory Unit, Maharaj Nakorn Chiang Mai Hospital, Chiang Mai, Thailand 50200

**Keywords:** GRACE risk score, STEMI patients with successfully fibrinolysis, Delay pharmacoinvasive strategy

## Abstract

**Background:**

Fibrinolytic therapy is the main reperfusion therapy for most STEMI patients in several countries. Current practice guidelines recommended routine early pharmacoinvasive (within 3–24 h after successful fibrinolysis, however it cannot be performed in timely fashion due to limitation of PCI-capable hospitals. This study aimed to evaluate the prognostic utility of the GRACE score in patients receiving delayed intervention after successful fibrinolysis in non PCI-capable hospital.

**Methods:**

We retrospectively analysed the data from the Maharaj Nakorn Chiang Mai Hospital acute ST-elevation myocardial infarction (STEMI) registry during the period 2007–2012. The STEMI patients who had successfully fibrionolysis in non PCI-capable hospital and received delayed PCI (during 24 h to 14 days after successful fibrinolytic therapy) at Maharaj Nakorn Chiang Mai hospital were included. The primary end point for this analysis was the composite outcomes, which included all-cause mortality, re-hospitalization with acute coronary syndrome (ACS), re-hospitalization with heart failure (HF) and stroke at 1 and 6-month.

**Results:**

A total of 152 patients were included. 88 patients and 64 patients were in low GRACE group (GRACE risk score ≤ 125) and intermediate to high GRACE group (GRACE risk score above 126), respectively. The median time from fibrinolysis to coronary intervention in low GRACE group was 8.5 days (interquartile range, 4.6–10.9) and 7.9 days (interquartile range,3.2,12.0) in intermediate to high GRACE group (*p* = 0.482). At 1 month, the composite cardiovascular outcome at 1 month occurred in 2 patients (2.3 %) in low GRACE group and 10 patients (15.6 %) in intermediate to high GRACE group (*P* = 0.003). During 6 months, the composite cardiovascular outcomes occurred in 6 patients (6.8 %) in low GRACE group and 12 patients (18.7 %) in intermediate to high GRACE group (*P* = 0.024). The cumulative of composite cardiovascular outcome was significant higher in intermediate to high GRACE group than in low GRACE group (Hazard ratio: 2.97, 95 % CI 1.11–7.90; *p* = 0.030).

**Conclusion:**

The long delay pharmacoinvasive strategy in intermediate to high GRACE score after successful fibrinolysis in non PCI-capable facilities were associated with worse cardiovascular outcomes than the patients with low GRACE score at 1 and 6 months. GRACE risk score may be helpful and guided the clinicians in non PCI-capable center in early transferred to early intervention in STEMI patients after fibrinolytic therapy.

**Electronic supplementary material:**

The online version of this article (doi:10.1186/s12872-016-0383-3) contains supplementary material, which is available to authorized users.

## Background

Primary percutaneous coronary intervention (PPCI) is preferred reperfusion therapy for acute ST-elevation myocardial infarction (STEMI). However, the PCI-capable center is still limited in several countries including Thailand. Therefore, fibrinolytic therapy is the main reperfusion therapy for most STEMI patients in our country. Current practice guidelines recommended routine coronary angiogram (CAG) after successful fibrinolysis, the so called pharmacoinvasive strategy [1–5]. However, early pharmacoinvasive (within 3–24 h after successful fibrinolysis) cannot be performed in a timely fashion due to limitation of PCI-capable hospitals. Previous acute coronary syndrome (ACS) registries, Thailand Registry in Acute Coronary Syndrome (TRACS) showed a low rate of coronary angiography and intervention during index admission and referral centers for early pharmacoinvasive strategy are still limited [6]. Therefore, risk stratification and identify risk of the patients are important in non PCI-capable hospital. Patients with intermediate to high risk for adverse cardiovascular event should be transferred for coronary angiogram as soon as possible. Although several risk scores for acute coronary syndrome (ACS) have been developed for stratified risk of ACS patients, GRACE (Global Registry of Acute Coronary Events) score is developed to focus on clinical risk assessment and to improve the selection of patients for clinical and interventional procedures following an ACS episode [7]. Many studies and meta-analysis demonstrated the accuracy and the usefulness of the GRACE score on the mortality of ACS patients in hospital and follow-up after hospital discharged [8–13]. This study aimed to evaluate the prognostic utility of the GRACE score in patients receiving delayed intervention after successful fibrinolysis.

## Methods

### Study design and population

We retrospectively analysed the data from the Maharaj Nakorn Chiang Mai Hospital STEMI registry during the period 2007–2012. The STEMI patients who had successfully fibrinolysis in non PCI-capable hospital and received delayed coronary intervention (during 24 h to 14 days after successful fibrinolytic therapy) at Maharaj Nakorn Chiang Mai hospital were included for analysis in the study. The exclusion criteria were the patients who unsuccessfully fibrinolysis (ST-segment decrease in elevation less than 50 % at 90 min after fibrinolysis), received early coronary intervention (<24 h after fibrinolytic therapy), received very delayed coronary intervention (>2 weeks after fibrinolytic therapy), the patients who denied for further interventions after fibrinolysis, the patients who received primary PCI or rescue PCI and the patients who had the previous history of coronary-artery bypass surgery. The protocol design was approved by the local institutional Research Ethics Committees of Faculty of Medicine, Chiang Mai University and Lampang hospital.

The data were collected from the medical recorded by the researcher. Demographic characteristics, medical history, presenting symptoms, baseline GRACE score time from symptom onset to administration fibrinolytic therapy, time from fibrinolysis to percutaneous coronary intervention, coronary intervention procedure and clinical outcomes were collected for analysis. In the patients who did not visit to the hospital to follow up, the telephone call was used to interview for evaluating the clinical outcomes.

### Definitions and end points

The STEMI was defined as the presence of at least 0.1-mV ST-segment elevation or new or presumably new left bundle branch block with elevation of cardiac enzyme levels above the reference range. Successfully fibrinolysis means the ST-segment decrease in elevation ≥ 50 % (partial resolution) and ≥ 70 % (complete resolution) at 90 min after fibrinolysis. Delayed coronary intervention means coronary intervention, including coronary angiogram and percutaneous coronary intervention performed during 24 h to 14 days after successfully fibrinolysis. The GRACE score composed of medical history (age, history of congestive heart failure, and history of myocardial infarction), findings at initial presentation (resting heart rate, systolic blood pressure, and ST-segment depression), and findings during hospitalization (initial serum creatinine, elevation of cardiac enzyme, and no in-hospital PCI), the total score range from 0–258 points. The patients were stratified into low (GRACE risk score <126), intermediate to high risk group (GRACE risk score ≥126). The primary end point for this analysis was composite outcomes, which included all-cause mortality, re-hospitalization with ACS, re-hospitalization with heart failure (HF) and stroke at 1 and 6-month. Re-hospitalized with ACS was defined as re-admission after discharge from hospital with ACS composed with clinical chest pain, rising of cardiac enzymes and dynamic ST-segment change. Re-hospitalized with heart failure was defined as re-admission after discharge from hospital with clinical decompensated heart failure or received intravenous diuretic. Culprit vessel PCI was defined as PCI confined to culprit vessel lesion only. The multivessel PCI was defined as PCI in which lesions in the culprit vessel as well as ≥1 non-culprit vessel lesions.

### Statistical analysis

Baseline demographics, procedural and angiographic characteristics presented with continuous measures and are expressed as mean ± standard deviation (SD) or median and interquartile range (IQR) wherever appropriate. The categorical data are expressed as number (percentages), except where indicated. Differences in continuous variables were analyzed with the Student’s t test or Wilcoxon rank-sum tests. The categorical variables were analyzed with Chi-square test and Fisher’s exact test. A *P*-value <0.05 was considered statistically significant. Composite endpoints and other clinical outcomes will be expressed as number (percentages). The prognostic utility of GRACE score on clinical outcomes was analyzed by logistic regression analysis and presented as odd ratio and area under the ROC curve (AuROC). The composite outcome was analyzed by use time to event analysis and presented with Kaplan-Meier curve. We conducted statistical analyses using Stata version 13 (Stata corporation, College Station, TX). The sample size was calculated by base on the data of the previous study of Yan et al. [14] reported death/myocardial re-infarction at 30 days in the standard treatment was 8.1 % and estimated 5 % loss of follow-up. To achieve a power of 80 %, with a type-1 error probability of 5 % (two-sided), allowable of estimated error (margin error) was 5 %, 120 patients were needed in this study.

## Results

### Baseline clinical characteristics

**Fig. 1:**
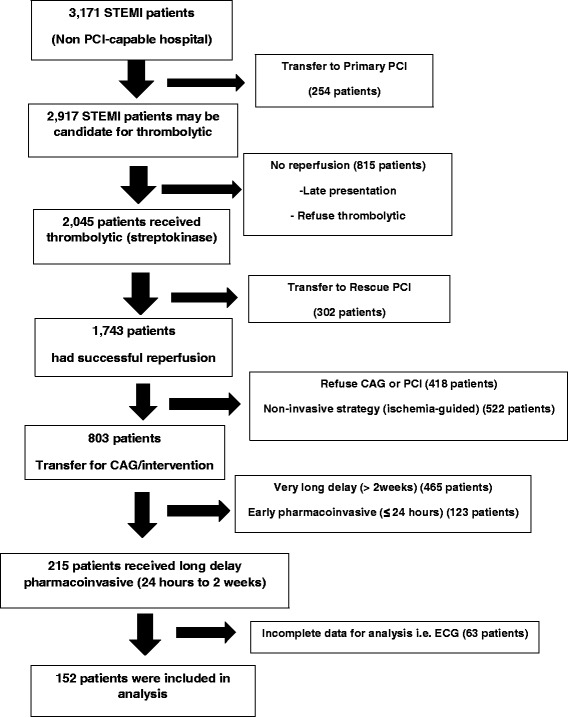
Study flow chart

Among 3171 STEMI patients during study period, 2045 STEMI patients received fibrinolytic therapy and a total of 152 patients met inclusion criteria, as shown in Fig. 1. Eighty-eight patients and 64 patients were in low GRACE group (GRACE risk score ≤ 125) and intermediate to high GRACE group (GRACE risk score above 126), respectively. The 6-month follow-up was available in 97 % of the patients in both groups. The clinical characteristics were shown in Table 1. The median time from fibrinolysis to coronary intervention in low GRACE group was 8.5 days (interquartile range, 4.6–10.9) and 7.9 days (interquartile range,3.2,12.0) in intermediate to high GRACE group (*p* = 0.482) (Additional file 1).

**Table 1:** Baseline clinical characteristics of patients according to GRACE risk score (*n* = 152)

Clinical characteristics	Low GRACE group (*N* = 88)	Intermediate to high GRACE group (*N* = 64)	*P*-value
Age (years), mean ± SD	55.3 ± 8.5	67.7 ± 3.2	< 0.001
Gender, (%)			0.696
Male	55(62.5)	38(59.4)	
Female	33(37.5)	26 (40.6)	
Time from symptoms onset to fibrinolysis, median (hours) (IQR:25th,75th percentile)	2.7 (IQR: 2,3.8)	2.8 (IQR: 2,4.5)	0.347
Time from fibrinolysis to CAG or PCI median (days) (IQR:25th,75th percentile)	8.5 (IQR:4.6,10.9)	7.9 (IQR: 3.2,12.0)	0.482
GRACE score, mean ± SD	100.2 ± 15.7	142.2 ± 13.4	< 0.001
LVEF (%)	54.9 ± 10.6	52.5 ± 13.6	0.239
Preexisting medical conditions, n (%)			
Diabetes	18 (20.4)	10.6 (15.6)	0.448
Hypertension	39 (44.3)	27 (42.2)	0.794
Dyslipidemia	30 (34.1)	16 (25.0)	0.228
Smoking	50 (56.8)	34 (53.1)	0.651
Chronic kidney disease	3 (3.4)	3 (4.7)	0.681

### Angiographic findings, procedural details and complications of the procedure

Angiographic findings and procedural details were presented in Table 2. Double vessel disease and triple vessel disease presented in 45.5 and 65.6 % in low GRACE group and intermediate to high GRACE group respectively. Lesion type B2 and C presented in 44.6 and 53.8 % in low GRACE group and intermediate to high GRACE group respectively. Sixty-three percent (*N* = 56) of the patients in low GRACE group and 61 % (*N* = 39) of the patients in intermediate to high GRACE group underwent PCI (*P* =0.738) while 36 % of the patients in low GRACE group (*N* = 32) and 39 % of the patients in intermediate to high GRACE group (*N* = 25) had only coronary angiography (*p* = 0.738). Culprit vessel PCI was performed in 89 % (*N* = 50) of the patients in low GRACE group and 92 % (*N* = 36) of the patients in intermediate to high GRACE group (=0.733). Among the patients underwent PCI, 76.8 % (*N* = 43) of the patients in low GRACE group and 76.3 % (*N* = 29) of the patients in intermediate to high GRACE group received drug-eluting stent (DES). The complications during and post procedure were shown in Table 3.

**Table 2:** Angiographic findings and procedural details (*n* = 152)

Angiographic findings and procedural details	Low GRACE group (*N* = 88)	Intermediate to high GRACE group (*N* = 64)	*P*-value
Angiographic findings, n (%)			
Mild disease	9 (10.2)	1 (1.6)	0.045
Single vessel disease	39 (44.3)	21 (32.8)	0.180
Double vessel disease	24 (27.3)	22 (34.4)	0.375
Triple vessel disease	16 (18.2)	20 (31.2)	0.082
Lesions (according to ACC/AHA), n (%)			
Type A	14 (25.0)	7 (18.0)	0.461
Type B1	17 (30.4)	11 (28.2)	0.503
Type B2	17 (30.4)	10 (25.6)	0.651
Type C	8 (14.2)	11 (28.2)	0.120
Procedural performed			
CAG without PCI, n (%)	32 (36.4)	25 (39.1)	0.738
Medical treatment	26 (81.2)	15 (60.0)	0.136
CABG	6 (18.8)	10 (40.0)	0.136
PCI, n (%)	56 (63.6)	39 (60.9)	0.738
Culprit vessel PCI	50 (89.3)	36 (92.3)	0.733
Multivessel PCI	6 (10.7)	3 (7.7)	0.733
Procedural details, n (%)			
POBA	1 (1.8)	6 (15.4)	0.018
Thrombus aspiration	0 (0)	3 (7.7)	0.066
Bare metal stent	13 (23.2)	5 (13.2)	0.290
Drug eluting stent	43 (76.8)	29 (76.3)	0.574

**Table 3:** Complications during and post-procedure (*n* = 152)

Complications	Low GRACE group (*N* = 88)	Intermediate to high GRACE group (*N* = 64)	*P*-value
During procedure, n (%)			
Abrupt vessel closure	0 (0)	0 (0)	NS
New thrombus formation	2 (3.57)	0 (0)	0.511
Side branch occlusion	0 (0)	0 (0)	NS
No reflow	2 (3.57)	0 (0)	0.511
Dissection	1 (1.8)	0 (0)	0.589
Emergency unplanned CABG	0 (0)	0 (0)	NS
Post procedure, n (%)			
Hematoma	0 (0)	0 (0)	NS
Hematuria	1 (1.1)	0 (0)	0.579
Gastrointestinal bleeding	0 (0)	0 (0)	NS
Required blood transfusion	0 (0)	0 (0)	NS
Contrast-induced nephropathy	0 (0)	0 (0)	NS

### Clinical outcomes

At 1 month, the composite cardiovascular outcome at 1 month occurred in 2 patients (2.3 %) in low GRACE group and 10 patients (15.6 %) in intermediate to high GRACE group (*P* = 0.003). During 6 months, the composite cardiovascular outcomes occurred in 6 patients (6.8 %) in low GRACE group and 12 patients (18.7 %) in intermediate to high GRACE group (*p* = 0.024) (Table 4). There was no death in hospital in low GRACE group when 2 patients (3.1 %) in intermediate to high GRACE group died (*P* = 0.176). Rate of re-hospitalized with HF at 1 and 6 months were significantly higher in intermediate to high GRACE group than low GRACE group (9.4 % vs 1.1 %, *p* = 0.022 and 10.9 % vs 2.3 %, *p* = 0.036, respectively).

**Table 4:** Clinical outcomes at 1 and 6 months of follow-up (*n* = 152)

Clinical outcomes	Low GRACE group (*N* = 88)	Intermediate to high GRACE group (*N* = 64)	*P*-value
In-hospital mortality, n (%)	0 (0)	2 (3.1)	0.095
At 1 month			
Composite outcomes	2 (2.3)	10 (15.6)	0.003
ACS	1 (1.1)	1 (1.6)	0.666
Heart failure	1 (1.1)	6 (9.4)	0.022
Stroke	0 (0)	1 (1.6)	0.421
CV death	0 (0)	2 (3.1)	0.180
Non-CV death	0 (0)	0 (0)	NS
Loss to follow-up	2 (2.3)	2 (3.1)	0.562
At 6 month (cumulative)			
Composite outcomes	6 (6.8)	12 (18.7)	0.024
ACS	4 (4.5)	1 (1.6)	0.298
Heart failure	2 (2.3)	7 (10.9)	0.036
Stroke	0 (0)	2 (3.1)	0.421
CV death	0 (0)	2 (3.1)	0.175
Non-CV death	0 (0)	0(0)	NS
Loss to follow-up	2 (2.3)	2 (3.1)	0.562

### The GRACE score and clinical outcomes

**Fig. 2:**
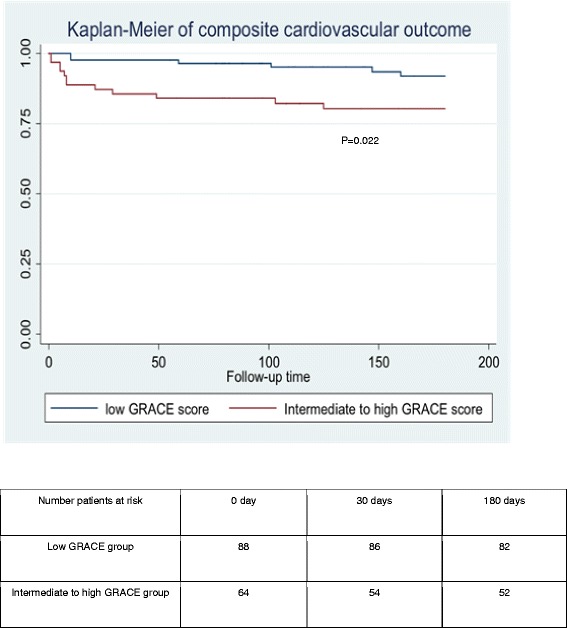
Kaplan-Meier of composite cardiovascular outcome

**Fig. 3:**
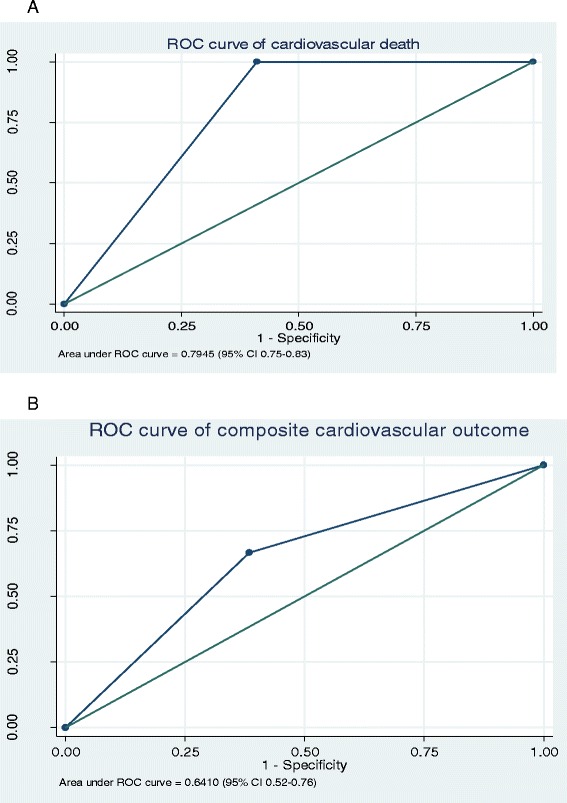
The Area under the Curve (AuROC) for the performance of GRACE score in predicting cardiovascular event. **a** 6-month cardiovascular death 6-month cardiovascular death (AuROC =0.794, 95 % CI 0.75–0.83). **b** Composite cardiovascular outcome (AuROC = 0.641, 95 % CI 0.52–0.76)

The composite cardiovascular outcome and re-hospitalized with HF at 6 months were higher in intermediate to high GRACE group than in the low GRACE group (OR: 3.20, 95 % CI: 1.13–9.06; *P* = 0.029 and OR: 5.34, 95 % CI: 1.07–26.68; *P* = 0.041 respectively). The cumulative of composite cardiovascular outcome was significant higher in intermediate to high GRACE group than in low GRACE group (Hazard ratio: 2.97, 95 % CI 1.11–7.90; *P* = 0.030), as shown in Fig. 2. We analysed the prognostic utility of GRACE score on clinical outcomes by the evidence from the area under the ROC curve. The area under the ROC (AuROC) of GRACE score for 6-month cardiovascular death was 0.794 (95 % CI 0.75–0.83). The AuROC of composite cardiovascular outcomes was 0.641 (95 % CI 0.52–0.76), as shown in Fig. 3.

## Discussion

Although early pharmacoinvasive strategy (within 3–24 h) after successful reperfusion are recommended by several guidelines [1–5], timely fashion CAG is not widely available in countries with limited PCI capable hospitals including Thailand. Several randomized trials and meta-analysis have shown that early routine post-thrombolysis angiography with subsequent PCI reduced the rates of re-infarction and recurrent ischemia compared with a watchful waiting strategy, in which angiography and revascularization were indicated only in the patients with spontaneous or induced severe ischemia or left ventricular (LV) dysfunction [15, 16]. The benefits of early routine PCI after thrombolysis were seen in the absence of increased risk of adverse events in many studies [15, 16]. The data from TRACS showed only half (50 %) of STEMI patients performed CAG on index admission. Fibrinolysis (especially streptokinase), is the first choice for treatment in low risk STEMI patients (42.6 % of STEMI patients received streptokinase and 1 % received Tenecteptase) [6]. Because of only one cardiac catheterization (during the period 2007–2012) in Northern Thailand (Maharaj Nakorn Chiang Mai Catheterization laboratory), the geographic and long distance of transfer and few of number of interventional cardiologists, primary PCI and early routine PCI after successful fibrinolysis were very difficult for this situation. Rescue PCI or primary PCI were performed in the patients who failed fibrinolytic therapy or cardiogenic shock at presentation. Hence, most of the STEMI patients in Thailand, especially in Northern of Thailand who successfully fibrinolytic therapy received the long delay coronary intervention (more than 24 h after fibrinolysis) and some of them received elective PCI or very long delayed intervention or elective PCI (after 2 weeks from successful fibrinolytic therapy) [6]. Several studies demonstrated the worst cardiovascular outcomes in the patients who received delay coronary intervention after thrombolysis [15–21]. The Southwest German Interventional Study in Acute Myocardial infarction (SIAM III) evaluated the effects of transfer early PCI (within 6 h after fibrinolysis) compared with delay PCI strategy (elective PCI 2 weeks after fibrinolysis) [17]. The early PCI showed significant reduction of primary end point (death, re-infarction, target lesion revascularization (TLR) and ischemic events) (HR: 0.61; 95 % CI 0.42–0.88, *p* = 0.008) and higher long term survival than delayed PCI (*p* = 0.057) [17]. Similarly to The Trial of Routine Angioplasty and Stenting after Fibrinolysis to Enhance Reperfusion in Acute Myocardial Infarction (TRANSFER-AMI) trial, showed that the patients who transfer from non-PCI center within 6 h after thrombolysis had fewer ischemic complications than standard treatment (delayed PCI) without increasing of major bleeding [18]. A meta-analysis showed mortality benefit at 30-day and 1 year of the STEMI patients with early transfer PCI after fibrinolysis as compared with ischemic-guided intervention (delayed PCI) [15, 16]. The NORwegian study on District treatment of ST-Elevation Myocardial infarction (NORDISTEMI) study also demonstrated a significant reduction in the composite cardiovascular outcome (death, re-infarction, stroke, or recurrent ischemia) at 1 year in the patients with immediate transferred to PCI following with thrombolysis as compared with the patients in conservative arm treatment (6 % vs 16 %, *p* = 0.01) [19]. Similarly to The Combined Abciximab RE-teplase Stent Study in Acute Myocardial Infarction (CARESS-AMI) study, a more conservative strategy (i.e. angiogram only in cases of failed thrombolysis) was associated with a worse clinical outcome than the strategy of angiogram and intervention (if indicated) in all cases following thrombolysis (composite of death, re-infarction and refractory ischemia at 30-day, 11 % vs 4 %, *p* = 0.004) [20]. From the previous data, no studies demonstrated of the benefit in the cardiovascular outcomes of the early and/or delay pharmacoinvasive strategies in STEMI patients who received streptokinase for treatment similar to our study. On the data from CARESS-AMI [20] and TRANSFER-AMI [18], The American College of Cardiology (ACC) and the American heart association (AHA) give a class IIa recommendations for high risk features (such as Kilip class >2, extensive ST-elevation, left ventricular ejection fraction (LVEF) <35 %, or hypotension) should be immediate transferred to PCI-capable facilities [3, 4]. The transfer of low and moderate risk STEMI patients to PCI-capable center received a class IIb recommendation. No available data showed the benefit outcome of early transferred for PCI in low and moderate risk patients.

Risk stratification of the STEMI patients were very important for the clinicians in non-PCI capable hospital to use to guide for judged and selected the STEMI patients for early invasive strategy. GRACE risk score, one of clinical risk score, has been shown to be a good risk stratification score in population with STEMI and NSTE-ACS. Several studies demonstrated the validation and the usefulness of GRACE score in stratified the STEMI patients for an early invasive management (AUC = 0.81; 95 % CI 0.80–0.82 for STEMI and AUC = 0.80; 95 % CI 0.74–0.89 for NSTE-ACS) [12]. The AuROC of 6-month mortality and the composite cardiovascular outcome of our study were 0.794 (95 % CI 0.75–0.83) and 0.641 (95 % CI 0.52–0.76). From our study, the GRACE score seem to be better performance in the cardiovascular mortality rather than the composite cardiovascular outcome of the patients with long delay pharmacoinvasive as similar as the previous study [12]. But the usefulness of GRACE score for predict the composite cardiovascular outcome is still unclear. A subgroup analysis of TRANSFER-AMI trial revealed the beneficial outcome of early pharmacoinvasive strategy only in patient with a low to intermediate GRACE risk score (<155), while the early invasive strategy was associated with worse outcome in high-risk patients (≥155) [14]. The pharmacoinvasive strategy was associated with a lower risk of death/re-MI in the low-intermediate GRACE risk group (HR = 0.52, 95 % CI 0.32–0.86, *p* = 0.010), but a higher risk of death/re-MI in the GRACE high-risk group (HR = 1.98, 95 % CI 1.06–3.67, *p* = 0.031) [14]. From this subgroup analysis from TRANSFER-AMI, risk score may also guide the best strategy to achieve and maintain myocardial reperfusion after administration of fibrinolytic therapy [14]. Similar to our study, the longer delay pharmacoinvasive strategy (24 h to 2 weeks after successful fibrinolysis) in non PCI-capable facilities may associate with the worst of composite cardiovascular outcome (death, re-hospitalized with ACS, re-hospitalized with HF and stroke) at 30-day and 6-month when compared with the patients with low GRACE score (15.6 % vs 2.3 % at 30 days, *p* = 0.003 and 16.7 % vs 6.8 % at 6 months, *p* = 0.024). Therefore, the patients with intermediate to high GRACE risk score should be early transferred to PCI-capable center after fibrinolytic therapy.

The in-hospital mortality and 6-month mortality of our study was lower than the previous registry (TRACS) because the difference in baseline patient characteristics, the severity of the patients and the number of the patients received of the percutaneous coronary intervention on admission (in-hospital mortality 5.3 % vs 3.1 % and 6-month mortality 12.1 % vs 3.1 %) [6]. Most of the patients in our study had multivessel disease but underwent culprit vessel PCI only in significant proportion of patients. A small number of the patients underwent multivessel PCI during index hospitalization (10.7 % in low GRACE group vs 7.7 % in intermediate to high GRACE group). The meta-analysis and systematic review of Moretti et al. [22] in management multivessel coronary disease in STEMI patients, 5855 patients from 6 studies (1 RCT) compared between only culprilt vessel PCI vs complete PCI performed during index hospitalization. No difference in major adverse cardiovascular events (MACE) at short-term (90 days) and long term outcome at 1 year but significant reduced the repeat revascularization at 1 year similar to culprit vessel PCI vs complete revascularization during PCI. The rate of CABG was high especially in intermediate to high GRACE group because of high prevalence of multivessel disease and complex coronary artery disease (Type B2 and C) which may suitable for CABG after acute phase of STEMI. Previous ACS registry in Thailand (TRACS) showed the lower rate of CABG but the revascularization data was collected only in hospital-phase of STEMI [6]. The selective biased in enrolled patients who survived during index admission may contribute to low cardiovascular event in our study. We showed the performance of GRACE score for mortality of in-hospital, short term (30 days) and 6-month Therefore, the GRACE risk score is useful for prediction in short- and long-term mortality of the STEMI patients with successful fibrinolysis and delay intervention in non PCI-capable hospital.

There are some limitations in our study that may compromise clinical implication. Our study was a retrospective observational study (non-randomized). The large number of excluded patients reflected the limited accessibility to coronary intervention within 2 weeks. The mortality was lower than the previous study because the small number of patients with high GRACE risk were included in our study.

## Conclusion

The long delay pharmacoinvasive strategy in intermediate to high GRACE score after successful fibrinolysis in non PCI-capable facilities were associated with worse cardiovascular outcomes (death, re-hospitalized with ACS, re-hospitalized with HF and stroke) than the patients with low GRACE score at 30 days and 6 months. GRACE risk score may be helpful and guided the clinicians in non PCI-capable center in early transferred to early intervention in STEMI patients after fibrinolytic therapy.

## Additional file

Additional file 1: Prognostic value of GRACE. The dataset for the prognostic value of GRACE risk score analysis. (XLSX 52 kb)

## Abbreviations

ACC: The American College of Cardiology
ACS: Acute coronary syndrome
AHA: The American heart association
AuROC: Area under receiver operating characteristic
BMI: Body mass index
CAG: Coronary angiography
CARESS-AMI: The combined abciximab RE-teplase stent study in acute myocardial infarction study
CV: Cardiovascular
GRACE score: The Global Registry for Acute Coronary Events score
HF: Heart failure
IQR: Interquartile range
LVEF: Left ventricular ejection fraction
MACE: Major adverse cardiovascular events
NORDISTEMI: The NORwegian study on District treatment of ST-Elevation Myocardial infarction study
NS: No statistical significant
NSTE-ACS: Non ST-elevation acute coronary syndrome
PCI: Percutaneous coronary intervention
PPCI: Primary percutaneous coronary intervention
RCT: Randomized control trial
ROC: Receiver operating characteristic
SD: Standard deviation
SIAM III: Southwest German Interventional Study in Acute Myocardial infarction study group
STEMI: ST- elevation myocardial infarction
TLR: Target lesion revascularization
TRACS: Thailand Registry in Acute Coronary Syndrome
TRANSFER-MAI trial: The trial of routine angioplasty and stenting after fibrinolysis to enhance reperfusion in acute myocardial infarction
